# A Pilot Cross-Sectional Study on the Level of Depression and Physical Activity among Students in Poland and Portugal in the Second Year of the COVID-19 Pandemic

**DOI:** 10.3390/jcm12072541

**Published:** 2023-03-28

**Authors:** Anna Zalewska, Monika Gałczyk, Marek Sobolewski, Hélder Fernandes

**Affiliations:** 1 Faculty of Health Sciences, Lomza State University of Applied Sciences, 14 Akademicka St., 18-400 Lomza, Poland; 2 Plant of Quantitative Methods, Rzeszow University of Technology, Al. Powstancow Warszawy 12, 35-959 Rzeszow, Poland; 3 Health Sciences Research Unit: Nursing (UICISA: E), Instituto Politécnico de Bragança, 5300-253 Bragança, Portugal

**Keywords:** physical activity, depression, COVID-19, students, Poland, Portugal

## Abstract

**Objectives:** The aim of the study was to make a preliminary assessment of depression rates and levels of physical activity and the association between physical activity and depression among Polish and Portuguese students in the second year of the COVID-19 pandemic. **Methods:** A web-based online survey was conducted among students in Poland and Portugal (398 respondents—229 from Poland and 169 from Portugal) aged between 17 and 26 in October 2022. The level of depression was assessed by the Beck Depression Inventory (BDI) in Polish and in Portuguese. The level of physical activity was measured by the International Physical Activity Questionnaire (IPAQ) in Polish and in Portuguese. **Results:** Overall, 20–35% of respondents suffered from moderate depression, with a lower proportion among men. There were no differences in the percentage distribution of depression classification between Polish and Portuguese students. A decrease in the severity of depression was observed with increasing physical activity. In both countries, walking more often had the strongest effect on reducing depression scores. **Conclusions:** The continued and alarming prevalence of depressive disorders among university students in the year after the pandemic began had a significant impact on this population—further research on the impact of physical activity on mental health in different populations should be conducted to adjust the optimal level of physical activity for alleviating negative psychiatric symptoms.

## 1. Introduction

Change in functioning in daily activities related to introduced learning and online work was related to the announcement of the COVID-19 pandemic in 2020. The governments of most countries implemented the measures recommended by the World Health Organization such as social distancing to limit the spread of coronavirus in the population [1,2]. Isolation for several months and staying indoors for most of the day forced not only children and adolescents but also adults to change their lifestyle from active to passive [3]. Restrictions on exercise, and bans on gyms, swimming pools, and health clubs have largely led to a decline in daily physical activity [4].

The World Health Organization defines physical activity as any movement of the human body that is stimulated by skeletal muscles and simultaneously requires an expenditure of energy. To maintain health, 150 min of moderate physical activity or 75 min of vigorous activity per week is recommended [5,6]. Regular moderate physical activity is beneficial for people of all ages, regardless of gender, fitness, and health status, and most importantly improves quality of life [7,8,9]. 

The positive effects of physical activity on the musculoskeletal, cardiovascular, or neuromuscular systems have been observed. Physical activity is also recognized as a factor that prevents and has a therapeutic effect on mental disorders. In addition, it is one of the ways to deal with anxiety or depression and to strengthen self-esteem [10,11]. Regular physical activity is considered a good way to manage stress and improve mood, and has a positive effect on brain function [12,13]. Even 10 min of physical activity such as walking, jogging, or cycling can improve mood [14]. Limiting physical activity can be one of the risk factors that contribute to diabetes, hypertension, and obesity, or have a negative impact on mental health [15,16,17]. 

There are numerous studies in the literature confirming the positive effects of physical activity on human mental health [3,18,19]. Even short periods of reduced physical activity require research by multidisciplinary teams into the factors that may cause it and the detrimental effects on the function of numerous systems and organs.

**Figure 1:**
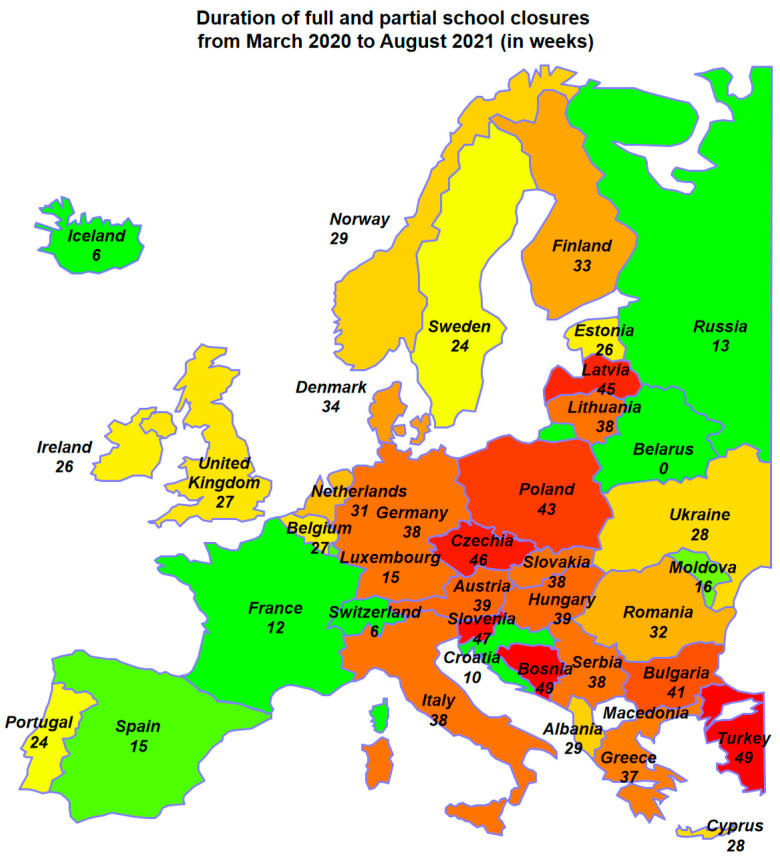
Duration of full and partial school closures in chosen Europe countries from March 2020 to August 2021 (Countries with a long period of school closures are marked with shades of red, and those with the shortest duration of distance learning are marked with shades of green.) [20].

Poland experienced one of the longest lockdowns in education in Europe. In Portugal, lockdowns lasted slightly shorter, placing the country in the middle, albeit among the top countries in Western Europe [20] (Figure 1). Consequently, the researchers decided to compare samples of the student populations from both countries.

The aim of the study was to make a preliminary assessment of depression rates and levels of physical activity as well as relationship between physical activity and depression among Polish and Portuguese students in the further year of the COVID-19 pandemic.

## 2. Materials and Methods

### 2.1. Participants and Procedure

In October 2022, students from Poland and Portugal participated in an online cross-sectional survey. The researchers distributed the survey by providing links to a Google form that asked students to participate in the survey on online e-learning platforms and on social media (Facebook) in private groups. The survey was accompanied by information about the study, anonymity, and voluntary consent to participate. In the groups for Polish students, the questionnaire was in Polish and in the groups for Portuguese students, the questionnaire was in Portuguese. Inclusion criteria were: student status, consent to participate in the study, residence in Poland or Portugal, full completion of the survey. Exclusion criteria were: no student status, no consent to participate in the study, no residence in Poland or Portugal, incomplete completion of the survey. A total of 502 responses to the researchers’ request were received. A total of 398 questionnaires were correctly completed and this group was analyzed. The study included 229 questionnaires from Poland and 169 from Portugal. The students polled ranged in age from 17 to 26 years. The study included 247 women and 151 men in total.

The Lomza State University of Applied Sciences Senate Committee on Ethics in Scientific Research gave its approval to the initiative. 

In accordance with Regulation (EU) 2016/679 of the European Parliament and of the Council of 27 April 2016, on the protection of natural persons with regard to the processing of personal data and on the free movement of such data and repealing Directive 95/46/EC, in the Personal Data Protection Act of 10 May 2018, participation in the study was voluntary, and the findings were published (Journal of Laws 2018, item 1000). GDPR, or the General Data Protection Regulation, the study’s goals, the poll’s methodology, and the relevant data protection rules were explained to the respondents.

### 2.2. Methods of Assessing the Level of Depression and Physical Activity

#### 2.2.1. Beck Depression Inventory

The level of depression was assessed using the Beck Depression Inventory (BDI) in Polish and in Portuguese. This is a questionnaire that consists of 21 questions and is used for self-assessment of the severity of depressive symptoms on a scale from 0 to 3 (further response options indicate increased symptom intensity). The level of depression is calculated after adding up the number of points. The summed measure of the Beck Depression Inventory can range from 0 to 63 points. Higher values obtained by the respondent indicate higher levels of depression [21]. Measures of depression are also considered on a 4-point scale, where: 0–11 means no depression; 12–26 means mild depression; 27–49 means moderate depression; 50–63 means severe depression [21]. The value for Cronbach’s alpha reported in papers is >0.7 [22,23,24,25].

#### 2.2.2. International Physical Activity Questionnaire

The level of physical activity was assessed using an abbreviated version of the International Physical Activity Questionnaire (IPAQ) in Polish and Portuguese, which is designed for people aged 15–69 years. It consists of 7 questions that cover all daily physical activities at work, in and around the home, and during leisure time. The questionnaire assesses activities that last continuously for at least 10 min. Each activity’s description is given in MET-min/week units, which are calculated by multiplying the activity’s coefficient by the number of days it was conducted throughout the week and by the activity’s duration in minutes per day [26,27]. In publications, Cronbach’s alpha is stated to be >0.7 [28,29,30]. 

### 2.3. Statistical Methods

For statistical analysis, Statistica v. 13 software was used (TIBCO Software Inc. (2017). Statistica (data analysis software system), version 13). The Mann–Whitney test was used to determine the significance of differences in depression severity (BDI) and physical activity level (IPAQ) between groups of Portuguese and Polish students, while the chi-square test of independence was used to determine differences in the percentage distribution of depression level classification and activity level. Spearman’s rank correlation coefficient was used to calculate correlations between depression severity and physical activity. The Kruskal–Wallis test was used to determine the significance of differences in depression severity (BDI) versus activity level classification. The non-normality of the distributions of the BDI and IPAQ measures, all of which had very strong right-handed asymmetry, dictated the use of non-parametric methods. A significance level of *p* < 0.05 (*) was established for all statistical analyses, but additionally denoted results for *p* < 0.01 (**) and *p* < 0.001 (***).

## 3. Results

### 3.1. General Characteristics

The analysis concerns a 398-member sample of students from Poland and Portugal. Both populations were predominantly female students—Poland 129 women (56.3%), Portugal 118 women (69.8%). The average age of the Polish and Portuguese students surveyed is similar (Table 1), so this is not a differentiating factor between the two study groups. They are comparable in this respect.

### 3.2. Comparison of Depression among Students in Both Countries by Gender 

Depression levels are generally lower among men, while there is no statistically significant difference in BDI values between the two countries—neither for men nor for women (Table 2).

After categorizing the BDI values into a four-point scale of depression severity (Table 3), it appears that the majority of students were not depressed (approximately 60–70% depending on gender and nationality). Moderate depression affected 20–35% of the respondents, while more severe depressive states occurred sporadically. There were also no differences between Polish and Portuguese students in the percentage distribution of depression classifications.

### 3.3. Comparison of Activities by Student Gender

The table (Table 4) provides detailed information on the measure of total physical activity, calculated from the IPAQ questionnaire. As gender is a differentiating factor in physical activity levels, the summary is given by female and male. No statistically significant differences were found in the physical activity levels of young people from the two countries, either for women or for men. For a measure showing a very high asymmetry in the distribution of results, as can be seen by comparing the mean (inflated by a few extremely high observations) and the median, a better measure of the average level is the latter figure.

The next table (Table 5) provides summaries for the detailed IPAQ measures and the overall measure in simplified form—the mean, median, and interquartile range (IQR) are provided. As can be seen, although there are no differences between countries on the overall measure, there is a significantly lower level of activity among Portuguese female students on the intensive effort measure (*p* = 0.0039 *) and to a lesser extent on the moderate effort measure (*p* = 0.0801).

When comparing the IPAQ measure classifications (Table 6) for the numeric IPAQ measure scores, the differences between the two countries are not statistically significant, while for the classification it seems that the Portuguese female students showed a different profile of physical activity—there were fewer of them with low activity (16% vs. 34%), but also with high activity (27% vs. 43%). Thus, the Polish women showed a more extreme attitude, while more than half of the Portuguese women showed a medium level of activity.

### 3.4. Depression and Physical Activity

It was investigated to what extent the level of physical activity was related to the severity of depression, considering different intensities. For this purpose, a correlation analysis was performed between the IPAQ scores and the BDI score. Due to the very pronounced influence of gender on IPAQ measures (they are significantly higher in men), the analysis was performed by gender and separately for both countries (Table 7).

In Portuguese women, there was no statistically significant correlation between activity and depression, while in Polish women the only statistically significant (*p* = 0.0155 *) but very weak correlation (*r*_S_ = −0.21) was between walking intensity and depression. The negative sign of the correlation means that more walking had a positive effect on the psyche, resulting in a minimal reduction in the severity of depression.

A stronger effect of physical activity on reducing depression was observed in men. The correlations were slightly stronger in the Polish population. In both countries, more frequent walking had the strongest effect on reducing BDI (in Poland, *r*_S_ = −0.42; in Portugal, *r*_S_ = −0.28).

Complementary to this analysis, information on the distribution of BDI in groups separated by physical activity level is summarized (Table 8). When the IPAQ is split into groups, some information is lost, and it is therefore more difficult to obtain a statistically significant relationship. In general, however, a weak trend toward a decrease in depression severity with increasing activity can be seen in the Polish group. However, because the differences in depression severity between groups with varying levels of physical activity were not statistically significant, the preceding observation should be regarded as a hypothesis for further research rather than a definitive conclusion. The *p*-value was less than 0.1 only for male students from Poland, indicating a difference close to statistical significance.

## 4. Discussion

It has been almost three years since the COVID-19 pandemic hit the world and negatively affected not only people’s physical health, but also their mental health [31]. Depression among university students proved to be one of the most important psychological consequences of the pandemic [32]. At the same time, students suffered at a higher rate compared to the general population [33]. Despite the existing literature on the psychological consequences of the pandemic and the health effects of physical activity, this issue is still relevant.

Results of a systematic review and meta-analysis conducted during the pandemic on the prevalence of depression among students worldwide showed that, on average, it affected 37% (95% CI, 32–42%) of the student population [31]. In a study at the beginning of the pandemic, up to 31% of students in Poland were found to suffer from moderate or severe depression, depending on where the study was conducted [34]. It was also observed that the number of depressions increased significantly as the pandemic progressed [35]. At the same time, data on the prevalence of depression among students in Portugal at the very beginning of the pandemic indicated levels in the range of 17.2% [36], then 24.2% [37]. However, other studies present higher levels of depressive disorders among Portuguese students and also an increased prevalence as the pandemic progressed [38,39]. In addition, 49.2% of Portuguese respondents surveyed by Mauro et al. reported moderate or severe psychological effects of the outbreak [40]. Although our own study found that the majority of students were not depressed, it was alarming that, in the second year of the pandemic, after most sanitary restrictions had been lifted and the vaccine COVID-19 had been released for general use, 20-35% of respondents were still affected by moderate depression.

In meta-analyses available in the literature, the most significant difference in levels of depression during the pandemic was found between geographical regions. Researchers conducting a review and meta-analysis of studies reporting levels of depression among dental students during the COVID-19 found a significantly higher prevalence of depression in studies from Asia compared to Europe and the Americas [32], which is confirmed by other meta-analyses [41,42]. Differences in levels of depression between different countries may be due to a number of factors, e.g., tools used to measure variables, sample size, the curricular load, and existing socio-cultural differences between countries [36]. Studies conducted at the very beginning of the pandemic on levels of depression in Switzerland and Portugal showed disadvantage of the Portuguese population, which has been linked, among other things, to cultural differences between countries [43]. 

At the same time, cumulative evidence from a meta-analysis on depression in Eastern Europe during the COVID-19 reveals high prevalence rates of clinically significant symptoms during the pandemic in Eastern Europe [44]. Psychiatric care in Eastern Europe depends on large psychiatric institutions with a focus on inpatient psychiatry, which is not effective in treating depression [45]. In Poland, lack of funding and easy and general access to specialized psychiatric and psychological care also contribute to poor mental health [46]. In Portugal, despite the high prevalence of mental disorders, available data suggest that a significant proportion of people do not receive adequate mental health care [47]. Despite the geographical location, there are no differences in the percentage distribution of depression classifications between Polish and Portuguese students, which may be related to similar problems in accessing mental health care in both countries. 

Previous long-term studies, conducted before the pandemic, had already found that women were more likely to develop depression, which is related to gender characteristics [48]. These findings are consistent with other studies conducted in the general population, which found higher rates of depression in women compared to men during the pandemic COVID-19 [49], as well as results from studies conducted in university students [50,51]. In our study, we found that depression rates were generally lower in men, while there was no statistically significant difference in BDI scores between the two countries.

Physical activity can effectively stimulate specific brain regions, thereby improving emotion and behavior regulation, promoting self-regulation of emotions, and helping to apply more adaptive emotion regulation strategies to better cope with stress during a pandemic [52,53]. Physical exertion also leads to a marked increase in sympathetic nervous system activity and catecholamine release. As a result, it may be able to regulate the secretion of melatonin, which significantly improves cardiovascular function, increases skeletal muscle adaptability, and protects body health [54]. Early in the pandemic, it was clear that increased physical activity had a positive effect on mental health in non-students, and regular maintenance of physical activity during the COVID-19 pandemic was associated with lower levels of anxiety and depression in subjects [2,18]. Our study also shows a tendency for depression severity to decrease with increased physical activity. A stronger effect of physical activity on reducing depression was observed in men. The correlations are slightly stronger in the Polish population, but it is difficult to clearly explain this difference.

Ongoing meta-analyses have shown that physical activity of different intensity significantly improves depressiveness and anxiety in university students [55,56]. In our study, a subgroup analysis showed that physical activity at a certain intensity could reduce students’ depressiveness. In both countries, more frequent walking had the strongest effect on reducing depression scores (in Poland, *r*_S_ = −0.42; in Portugal, *r*_S_ = −0.28). These results are consistent with the conclusions drawn on the basis of studies conducted during the pandemic. It was concluded that moderate-intensity training may be the optimal training intensity for promoting mental health by reducing TNF-α. This has important implications for dosing recommendations for physical activity for the treatment of mental illness [57]. However, there is no definitive consensus in the literature on the optimal level of physical activity to alleviate negative psychiatric symptoms [11]. There are other studies, indicating that it is, for example, intensive (6 < 9 Mets) physical activity that should be superior to moderate (3 < 6 Mets) physical activity in the treatment of people with depressive disorders [55].

There were no statistically significant differences in physical activity levels among adolescents from the two countries, either in women or men. Portuguese female students showed a different physical activity profile, with fewer low-activity (16% vs. 34%) but also high-activity (27% vs. 43%) students. There were more extreme attitudes among Polish women, while more than half of Portuguese women were active at an intermediate level. Similar results were obtained by Król et al. who studied physical activity levels among students in Poland, Portugal, and Belarus [58].

The present study has several limitations that should be noted. First, the cross-sectional nature of the study does not provide robust and causal evidence for the observed associations. The second limitation was the small sample group and preliminary findings. The study was also conducted in an online survey format and the identity of those who responded is not known [59]. Social media particularly attracts stressed individuals looking for support, which may distort the data and affect the representativeness of the sample. All data were collected using self-report questionnaires, which may bias responses [60]. Despite these limitations, the current study has a number of strengths, such as its potential contribution to the field of mental health, as it presents mental health and physical activity outcomes for the second year of the pandemic. The use of standardized and validated instruments was also a strength of the present study, as well as the easy access to the study group, low cost, and small amount of time spent on the project. Future studies should target a larger sample and use more objective methods to assess the parameters studied.

## 5. Conclusions

Considering the persistent and alarming prevalence of depressive disorders among students in the next year of exposure to the SARS-CoV-2 virus, this situation can be expected to have a significant impact on this population. Understanding the prevalence of mental disorders in specific regions of Europe and the factors that have a positive impact on them can help to create targeted health policies. Further research should be conducted on the impact of physical activity on mental health in different populations, with the aim of adjusting the optimal level of physical activity for the alleviation of negative mental symptoms. Pilot studies have shown that there are not many statistically significant differences in depression and physical activity levels between students in Poland and Portugal.

## Figures and Tables

**Table 1:** Average age of respondents.

Country	Age (Years)						
	Mean	Me	Std. Dev.	*c*_25_	*c*_75_	Min	Max
Poland	22.1	22	1.7	21	23	18	26
Portugal	21.6	22	1.8	21	22	17	26

**Table 2:** Measures of depression severity in Poland and Portugal by gender.

Country	BDI Measure of Depression Severity									
	Female (*p* = 0.3418)					Male (*p* = 0.6034)				
	Mean	Me	Std. Dev.	c_25_	*c*_75_	Mean	Me	Std. dev.	c_25_	*c*_75_
Poland	9.7	7	10.1	1	18	7.2	4	8.6	0	12
Portugal	8.9	4	10.6	0	15	7.9	2	11.3	0	15

*p*—test probability value calculated by the Mann–Whitney test.

**Table 3:** Level of depression severity in Poland and Portugal according to gender on a four-point scale.

Level of Depression	Gender			
	Female (*p* = 0.3660)		Male (*p* = 0.7722)	
	Poland	Portugal	Poland	Portugal
no depression	80 (62.0%)	81 (68.6%)	73 (73.0%)	37 (72.5%)
moderate	43 (33.3%)	30 (25.4%)	23 (23.0%)	10 (19.6%)
severe	5 (3.9%)	7 (5.9%)	4 (4.0%)	4 (7.8%)
very severe	1 (0.8%)	0 (0.0%)	0 (0.0%)	0 (0.0%)

*p*—test probability value calculated by means of the chi-square independence test.

**Table 4:** Measure of total physical activity.

Country	IPAQ Total Effort (MET-min./Week)									
	Female (*p* = 0.3731)					Male (*p* = 0.8920)				
	Mean	Me	Std. Dev.	c_25_	*c*_75_	Mean	Me	Std. Dev.	c_25_	*c*_75_
Poland	4960	1800	5631	582	8136	6118	4491	5266	1598	9222
Portugal	3791	1382	5540	742	4068	7534	4110	7437	1022	13,050

*p*—test probability value calculated by the Mann–Whitney test. IPAQ—International Physical Activity Questionnaire. MET—Metabolic Equivalent Task.

**Table 5:** Summary of IPAQ specific measures.

IPAQ Measures (MET-min./Week)	Poland			Portugal			*p*
	Mean	Median	IQR	Mean	Median	IQR	
Women							
Intense effort	2247	480	3600	1533	0	1440	0.0039 **
Moderate effort	1324	480	1920	938	400	840	0.0801
Walking	1390	594	2178	1320	693	924	0.2052
Total effort	4960	1 800	7554	3791	1382	3326	0.3731
Men							
Intense effort	2961	2400	3840	3511	2400	6720	0.8491
Moderate effort	1712	960	2090	2198	960	3160	0.7808
Walking	1445	891	1832	1824	1040	2475	0.1652
Total effort	6118	4491	7624	7534	4110	12,028	0.8920

*p*—test probability value calculated using Mann–Whitney test, *p* < 0.01 (**). IQR—Interquartile Range.

**Table 6:** Summary of IPAQ measure classifications.

Physical Activity Level	Gender			
	Female (*p* = 0.0000 ***)		Male (*p* = 0.6395)	
	Poland	Portugal	Poland	Portugal
low	44 (34.1%)	19 (16.1%)	11 (11.0%)	7 (13.7%)
average	30 (23.3%)	67 (56.8%)	30 (30.0%)	18 (35.3%)
high	55 (42.6%)	32 (27.1%)	59 (59.0%)	26 (51.0%)

*p*—test probability value calculated by means of the chi-square independence test, *p* < 0.001 (***).

**Table 7:** Correlation coefficients between physical activity 19 and the BDI depression index.

IPAQ	BDI	
	Poland	Portugal
Women		
Intense effort	−0.05 (*p* = 0.5432)	−0.03 (*p* = 0.7843)
Moderate effort	−0.15 (*p* = 0.0800)	−0.09 (*p* = 0.3436)
Walking	−0.21 (*p* = 0.0155 *)	−0.08 (*p* = 0.3878)
Total effort	−0.12 (*p* = 0.1664)	−0.06 (*p* = 0.5020)
Men		
Intense effort	−0.11 (*p* = 0.2908)	−0.24 (*p* = 0.0924)
Moderate effort	−0.24 (*p* = 0.0173 *)	−0.24 (*p* = 0.0867)
Walking	−0.42 (*p* = 0.0000 ***)	−0.28 (*p* = 0.0434 *)
Total effort	−0.22 (*p* = 0.0253 *)	−0.23 (*p* = 0.1066)

Spearman coefficient of correlation (with assessment of its statistical significance), *p* < 0.05 (*), *p* < 0.001 (***). BDI—Beck’s Depression Inventory. IPAQ—International Physical Activity Questionnaire.

**Table 8:** Distribution of BDI in groups separated by physical activity level.

Country/ Gender		Depression Severity (BDI) versus Physical Activity Level (IPAQ)												*p*
		Low				Average				High				
		*N*	Mean	Me	IQR	*N*	Mean	Me	IQR	*N*	Mean	Me	IQR	
Poland	women	44	11.5	10	16.5	30	8.9	7	10.0	55	8.7	5	18.0	0.2011
	men	11	13.0	16	17.0	30	6.4	2	7.0	59	6.6	4	12.0	0.0813
Portugal	women	19	11.9	11	19.0	67	6.7	2	11.0	32	11.5	5	21.0	0.1665
	men	7	7.9	9	15.0	18	7.9	2.5	8.0	26	7.9	0	20.0	0.1958

*p*—test probability value calculated using the Kruskal–Wallis test. BDI—Beck’s Depression Inventory. IPAQ—International Physical Activity Questionnaire.

## Data Availability

The data that support the findings of this study are openly available in RepOD at https://doi.org/10.18150/0J3FKR (accessed on 14 February 2023).
